# Preoperative smoking cessation program in patients undergoing intermediate to high-risk surgery: a randomized, single-blinded, controlled, superiority trial

**DOI:** 10.1186/s13063-022-06628-8

**Published:** 2022-08-29

**Authors:** Christian D. Fankhauser, Andres Affentranger, Beatrice Cortonesi, Urs Jeker, Markus Gass, Fabrizio Minervini, Georg Jung, Corina Christmann, Christine Brambs, Milo A. Puhan, Ulrike Held

**Affiliations:** 1 Epidemiology, Biostatistics and Prevention Institute, University of Zurich, Zurich, Switzerland; 2 University of Lucerne, Lucerne, Switzerland

**Keywords:** Randomized controlled trial, Smoking cessation, Surgery, Complications

## Abstract

**Background:**

At present, effectively implementing smoking cessation programs in the health care system constitutes a major challenge. A unique opportunity to initiate smoking cessation focuses on smokers scheduled for surgery. These patients are not only highly motivated to quit smoking but also likely to benefit from a reduction in postoperative complications which may translate into a decrease of costs. Nevertheless, surgical patients are not routinely informed about the benefits of preoperative smoking cessation. Potential reasons for this missed opportunity may be the lack of time and training of surgeons and anaesthesiologists. We therefore aim to analyse the impact of a preoperative high-intensity smoking cessation intervention on surgical complications up to a 90-day postoperative period in patients of various surgical disciplines. The hypothesis is that a preoperative smoking cessation program improves outcomes in smokers undergoing intermediate to high-risk surgery.

**Methods:**

The present study is a single-centre, randomized trial with two parallel groups of smokers scheduled for surgery comparing surgery alone and surgery with preoperative smoking cessation. We plan to randomize 251 patients. The primary objective is to compare complications between patients with an institutional multifaceted smoking cessation intervention starting 4 weeks before surgery compared to patients in the advice-only group (control group) within a 90-day postoperative period. The primary endpoint is the Comprehensive Complication Index (CCI®) within 90 days of surgery. Secondary outcomes include the length of hospital stay, cost of care, quality of life, smoking abstinence, and reduction in nicotine consumption.

**Discussion:**

The hypothesis is that a preoperative smoking cessation program improves outcomes in smokers undergoing surgery.

**Trial registration:**

BASEC #2021-02004; ClinicalTrials.gov: NCT05192837. Registered on January 14, 2022.

**Supplementary Information:**

The online version contains supplementary material available at 10.1186/s13063-022-06628-8.

## Administrative information

Note: the numbers in curly brackets in this protocol refer to SPIRIT checklist item numbers. The order of the items has been modified to group similar items (see http://www.equator-network.org/reporting-guidelines/spirit-2013-statement-defining-standard-protocol-items-for-clinical-trials/).

**Table:** 

Title {1}	Preoperative smoking cessation program in patients undergoing intermediate to high-risk surgery: a monocentric, randomized, single-blinded, controlled trial
Trial registration {2a and 2b}.	BASEC #2021-02004; Registration on Clinical Trials: NCT05192837 https://clinicaltrials.gov/ct2/show/NCT05192837 on January 14, 2022
Protocol version {3}	Version 3, 01/05/2022.
Funding {4}	This study is funded by the Swiss Cancer League (HSR-5217-11-2020) and regular reporting is required but investigators are analyzing the data independently.
Author details {5a}	Christian D Fankhauser^1, 2^, Andres Affentranger^1^, Beatrice Cortonesi^1^, Urs Jeker^2^, Markus Gass^2^, Fabrizio Minervini^2^, Georg Jung^2^, Corina Christmann^2^, Christine Brambs^2^, Milo A. Puhan^1^, Ulrike Held^1*^ ^1^ Epidemiology, Biostatistics and Prevention Institute, University of Zurich, Zurich, Switzerland ^2^ University of Lucerne, Lucerne, Switzerland *corresponding author
Name and contact information for the trial sponsor {5b}	PD Dr. med. Christian Fankhauser Spitalstrasse 6000 Lucerne Switzerland christian.fankhauser@luks.ch +41 41 205 11 11
Role of sponsor {5c}	Dr. Fankhauser is responsible for design; collection, management, analysis, and interpretation of data; writing of the report; and the decision to submit the report for publication and has the ultimate authority over any of these activities.

## Introduction

### Background and rationale {6a}

One quarter of the Swiss population smokes daily [1]. Smoking is one of the most important and few modifiable risk factors associated with cardiovascular or pulmonary diseases and cancer. Therefore, smoking cessation represents an important intervention in health care [2, 3]. At present, effectively implementing smoking cessation programs in the health care system constitutes a major challenge. A unique opportunity to initiate smoking cessation focuses on smokers scheduled for surgery. These patients are not only highly motivated to quit smoking [4] but also likely to benefit from a reduction in postoperative complications of 40% which may translate into a decrease of costs by 10% [5–7]. Nevertheless, surgical patients are not routinely informed about the benefits of preoperative smoking cessation. Potential reasons for this missed opportunity may be the lack of time and training of surgeons and anesthesiologists [8].

A systematic Cochrane review including 13 trials [7] concluded that preoperative smoking interventions increase short-term smoking cessation and reduce postoperative complications. The strongest signal was observed in two studies which performed an “intense smoking cessation program” including weekly face-to-face or telephone counselling over a period of at least 4 weeks prior to surgery. However, previous studies only reported postoperative complications up to a 30-day postoperative period and complications were not graded by severity. Additionally, most studies were performed in orthopaedic departments and therefore limiting the generalizability of its results.

We therefore aim to analyse the impact of a preoperative high-intensity smoking cessation intervention on surgical complications up to a 90-day postoperative period in patients of various surgical disciplines. Further advantages of our protocol represent the throughout coding and definition of postoperative complications and institutional approach. In this trial, we will report complications according to the Comprehensive Complication Index (CCI®) [9], which represents the most widely used and accepted measure of post-operative complications. Thereby, we improve reporting of postoperative morbidity since complications are graded by severity and the cumulative burden from any combination of complications is described in a single patient. Furthermore, our study will assess mid- to long-term smoking abstinence during follow-up to assess the impact of a preoperative non-physician-triggered smoking cessation program from a public health perspective.

### Objectives {7}

#### Hypothesis and primary objective

The hypothesis is that the preoperative smoking cessation program improves outcomes in smokers undergoing surgery. The primary objective is to compare complications between patients with an institutional multifaceted smoking cessation intervention compared to patients in the advice-only group (control group) within a 90-day postoperative period.

### Trial design {8}

This investigation is a monocentric, randomized, single-blinded, controlled superiority trial involving patients undergoing intermediate and high-risk surgery [10] at the Hospital of Lucerne. Patients will be randomised in a 1:1 ratio to either the intervention or control group. Before randomisation patients will be pre-stratified for age (≤60, >60 years) and procedure (intermediate versus high-risk procedures). Minimization, as an efficient way to control for confounding in small to moderately sized trials, will be used. Also, minimization automatically ensures the concealment of random allocation since there is no pre-existing randomization list.

## Methods: Participants, interventions, and outcomes

### Study setting {9}

Major urban hospital in Lucerne, Switzerland

### Eligibility criteria {10}

#### Inclusion criteria

- Patient listed for intermediate or high-risk surgery (Additional file 2) [10] at the Cantonal Hospital of Lucerne
- Patient undergoing surgery in one of the following departments: Abdominal surgery, thoracic surgery, urology, gynaecology, vascular surgery or head and neck surgery
- Date of surgery >4 weeks planned after date of listing for surgery or discussion by tumor board
- Current smokers, defined as daily smoking of at least one cigarette, cigar, or pipe
- Age ≥ 18 years
- Able to give signed written informed consent

#### Exclusion criteria

- Consumption of illegal drugs
- Alcohol dependency defined as preexisting alcohol-related disorders (eg. alcoholic psychosis, alcohol abuse, alcohol polyneuropathy, degeneration of the nervous system due to alcohol, alcoholic myopathy, alcoholic liver disease)
- Inability to follow the procedures of the study, e.g. due to language problems, psychological disorders, dementia

### Who will take informed consent? {26a}

Patients will be contacted and informed about the study over the phone. The study nurse will explain to each participant the nature of the study, its purpose, the procedures involved, the expected duration, the potential risks and benefits and any discomfort it may entail. Each participant will be informed that the participation in the study is voluntary and that he or she may withdraw from the study at any time and that withdrawal of consent will not affect his or her subsequent medical assistance and treatment. The participant will be informed that his or her medical records may be examined by authorised individuals other than their treating physician. All participants interested in the study will be provided a participant information sheet and a consent form describing the study and providing sufficient information for the participant to make an informed decision about their participation in the study.

### Additional consent provisions for collection and use of participant data and biological specimens {26b}

No samples will be stored and therefore no additional consent is required.

## Interventions

### Explanation for the choice of comparators {6b}

Patients randomised to the control arm will get advice only. Their preoperative course will be as if they were not participating in this study, meaning they will receive inconsistent perioperative smoking cessation advice from nurses, surgeons, or anaesthesiologists but no further study-specific smoking cessation intervention. Importantly, participants in the control group will not be discouraged from using perioperative smoking cessation aids and can still obtain help on their own initiative.

### Intervention description {11a}

The study intervention consists of an interview by a Tobacco Treatment Specialist (TTS) 4 weeks before surgery with individual counselling and offered nicotine substitution. All TTS have > 15 years of experience and underwent a postgraduation course for counselling smoking cessation. The intervention meeting is based on the Chronic Care Model (CCM) [11, 12] to improve the quality of ambulatory care through six interrelated system changes [13]: self-management support, decision support, delivery system design, clinical information systems, health care organisation, and community resources. In line with the CCM, the intervention will include the following elements adapted from Haas et al. randomized clinical trial [14] and are described in the treatment plan. In brief, the intervention includes:

- Informative content about advantages of smoking cessation [15] using information leaflets along with decision support
- Scheduling service for a motivational interview by a TSS in the office to provide educational and motivational content, explain the magnitude and profile of nicotine dependence and implement a treatment plan [16]
- For participants willing to quit smoking a preoperative quit day will be scheduled after the first intervention meeting 2–3 weeks before surgery. Time of a preoperative quit day may vary between patients, since our smoking intervention focuses on as early quit attempts as possible. Therefore, there will be a variety in patients with one or more quit attempts, with or without success, and patients who quit smoking in the postoperative period, only.
- Patients will be encouraged by a TSS to use nicotine replacement with patches/gums/pills at their own discretion. For smokers of 20 cigarettes per day or more, a 4-week supply of 21mg/day, 2-week supply of 14 mg/day and a 2-week supply of 7 mg/days patches will be provided. Patients who smoke between 10 and 20 cigarettes per day will receive a 4-week supply of 14 mg/day and a 4-week supply of 7 mg/day. Smokers of <10 cigarettes per day will be supplied with a 4- to 8-week supply of 7-mg/day patches. Bupropion or Varenicillin will be provided on an individual basis (all costs are covered by the patient’s insurance).

### Criteria for discontinuing or modifying allocated interventions {11b}

Patient preference.

### Strategies to improve adherence to interventions {11c}

TSS will schedule repeated follow-up meetings to support smoking abstinence or nicotine reduction before and after surgery for all patients. However, patients not interested in further smoking cessation counselling will not be forced to participate in additional follow-up meetings.

The patient’s general practitioners will be informed about the aim of the trial by e-mail and will be asked to further support the patient regarding smoking cessation and encourage abstinence at the end of follow-up as tobacco dependence might be better viewed as a chronic disorder, requiring repeated episodes of treatment.

### Relevant concomitant care permitted or prohibited during the trial {11d}

No further concomitant care is prohibited.

### Provisions for post-trial care {30}

TSS will schedule repeated follow-up meetings to support smoking abstinence or nicotine reduction before and after surgery for all patients. However, patients not interested in further smoking cessation counselling will not be forced to participate in additional follow-up meetings.

### Outcomes {12}

The primary endpoint is the (CCI®) [9] within 90 days of surgery. The CCI is calculated as the sum of all Clavien–Dindo complications [17] (Additional file 1) that are weighted for their severity (multiplication of the median preference values from patients and physicians). The final formula yields a continuous scale that ranks the cumulative burden from any combination of complications from 0 to 100 in a single patient. As a composite complication score, the CCI has the advantage of reflecting the overall burden of the postoperative course that affects the health of patients and their quality of life. In addition, the CCI is a powerful endpoint in trials, as it allows sample size up to nine times lower compared with traditional morbidity endpoints [18]. Complications will be assessed from medical health records by an advanced nurse practitioner supported by a surgical resident and consultant, all of whom were already trained in the Clavien–Dindo classification in a pilot study. Outpatient information of complications treated at other institutions will be included.

Secondary endpoints are length and costs of hospital stay, readmission rates for inpatient hospital stay, smoking abstinence or nicotine reduction, nicotine dependence, mental health, quality of life, unplanned postoperative intermediate care or intensive care unit admissions and cost and. Secondary endpoints will be recorded up to a 12-month postoperative follow-up period and will be compared between the intervention and control group. Smoking abstinence or nicotine reduction will be assessed by measuring smoking status (cigarettes smoked per day) and nicotine dependence will be assessed using the Fagerström test [19]. Nicotine abstinence will be additionally confirmed by using the NicAlert cotinine saliva test. Quality of life will be assessed with the German version of the 36-Item Short Form Survey (SF-36; index values range from 0 to 1, with higher scores indicating better quality of life) including covering physical functioning, bodily pain, role limitations due to physical health problems, role limitations due to personal or emotional problems, emotional well-being, social functioning, energy/fatigue, and general health perceptions during the past 4 weeks [20]. Mental health will be assessed using the Hospital anxiety and depression scale (HADS) [21].

Morbidity may influence the endpoints, therefore Charlson Comorbidity Index [22] and the American Society of Anaesthesiologists (ASA) physical status classification [23] will be assessed for each patient at baseline. The following characteristics measured at baseline are further described: age, gender, surgical procedure, underlying medical conditions and the stage of the Transtheoretical Model (TTM) of behaviour change.

### Participant timeline {13}

**Fig. 1:**
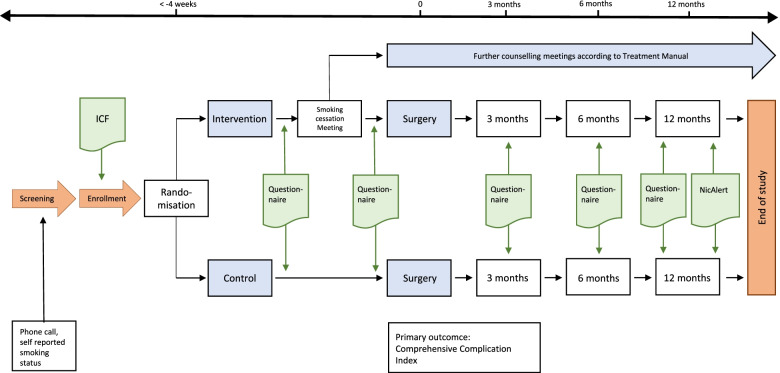
Participant timeline. ICF, informed consent form; NicAlert, nicotine saliva test

Fig. 1 shows the participant timeline.

### Sample size {14}

Based on a previous Cochrane review with meta-analysis [7], it is assumed that preoperative smoking cessation decreases the binary outcome of postoperative complications (yes or no) with a relative risk of 0.42. The mean postoperative CCI as a continuous measure in a retrospective chart review in a cohort without smoking cessation at the hospital of Lucerne was 13 and we therefore assume a CCI of 5.5 in the intervention group. The assumed standard deviation for the sample size calculation was 20. With anticipated 80% power, a two-sided significance level α of 5%, a sample size of 226 patients is planned. With an additional 10% dropout, our aim is to enrol 251 patients.

### Recruitment {15}

All patients scheduled for an intermediate or high-risk procedure (Additional file 2) at the hospital of Lucerne will be identified by the study nurse either through the tumour board lists or as soon as they are scheduled for surgery in the electronic health record program. Otherwise, clinicians can contact the study nurse to enrol patients who have been referred but not yet scheduled for surgery or presented at the multidisciplinary tumour board. To avoid an overload of the consulting capacities in the intervention arm recruitment of new trial participants may be paused.

## Assignment of interventions: allocation

### Sequence generation {16a}

Patients will be randomised in a 1:1 ratio to either the intervention or control group using the service of the University of Graz (www.randomizer.at). Before randomisation patients will be pre-stratified for age (≤60, >60 years) and procedure (intermediate versus high-risk procedures). Both factors have been identified in a previous retrospective cohort study at our institution as risk factors for complications. Minimization, as an efficient way to control for confounding in small to moderately sized trials, will be used. Also, minimization automatically ensures the concealment of random allocation since there is no pre-existing randomization list.

### Concealment mechanism {16b}

Minimization ensures concealment of random allocation since there is no pre-existing randomization list.

### Implementation {16c}

Upon recruitment of a new patient, the information on the two strata will be entered through a web browser, and the new treatment allocation is provided instantaneously.

## Assignment of interventions: Blinding

### Who will be blinded {17a}

Deidentified reports will be used for the assessment of the primary outcome of perioperative complications.

### Procedure for unblinding if needed {17b}

Unblinding for the primary outcome will not be necessary.

## Data collection and management

### Plans for assessment and collection of outcomes {18a}

Throughout the study, data is documented and collected in the electronic patient chart called LUKiS, a local installation of the CMR-System by the US-based Healthcare IT-System provider Epic. Next to the clinical part, the CMR system has a dedicated research-focused module (Epic Research) to aid researchers and their associated staff in facilitating research projects by providing tailored security, population selection, patient recruitment, data collection etc.

### Plans to promote participant retention and complete follow-up {18b}

The study nurse will follow up on all patients and collect postoperative patient-reported outcomes whenever possible.

### Data management {19}

Throughout the study, patient data is collected in LUKiS in an uncoded manner; study participants will be informed accordingly. At the end of the study, the LUKiS reporting team will generate a report, which will include all study data in a de-identified form. This means that participants cannot be identified in the report by name, initials, or birth date. An identification number (code), generated by LUKiS, will be used instead. The report and the list with the identification numbers will be provided to the study team. Both the dedicated LUKiS team as well as the study team have access to the report as well as to the list with the identification numbers. Both departments store the list with the identification numbers independently from the report.

### Confidentiality {27}

The analysis of the report with the pseudonymized data will be performed by the project statistician. None other than the study staff will have access to the de-identified report. The investigator and all study staff will maintain all study documents in strict confidence.

### Plans for collection, laboratory evaluation and storage of biological specimens for genetic or molecular analysis in this trial/future use {33}

Nicotine abstinence will be confirmed by using the NicAlert saliva test. The test will be performed by an advanced nurse practitioner during a routine hospital visit of the participant. The NicAlert saliva test is a point of contact test detecting six ranges of cotinine concentrations from 0 to 2000+ ng/ml. The only data collected is the category of concentration. No biological samples will be stored, and no further laboratory tests will be carried out. The test kit and the saliva sample will be immediately destroyed after use.

## Statistical methods

### Statistical methods for primary and secondary outcomes {20a}

A detailed statistical analysis plan has been written up as an Additional file 3 to the submission. According to the intention to treat (ITT) principle, patients will be analysed according to the treatment group they have been assigned to by randomisation. The full analysis set (FAS) consists of the patients randomized and receiving surgery within 4 months after randomization. The FAS will be the primary population for the efficacy analysis.

Primary outcome CCI will be addressed with a multiple linear model, including a randomized treatment group, and the minimization variables (age ≤60 or >60 and intermediate or high-risk surgery) as independent variables. If after normality check with a qq-plot the residual distribution is skewed a transformation of the CCI may become necessary. To evaluate the between-group difference in CCI of the smoking cessation intervention versus the control group, adjusted for the above-defined confounders, the estimated between-group difference with 95% confidence interval and corresponding p-value will be reported. For the secondary outcomes, linear, logistic, or Poisson regression models including the randomized treatment group as an independent variable, will be used to estimate the treatment effect between treatment groups. Normality will again be checked using qq-plot and, if necessary, appropriate transformations of the dependent variable will be made.

For the comparison of readmission rates between the treatment groups, the date of discharge from the hospital after surgery will be chosen as the starting observational time point and a Poisson model, considering readmissions collectively, will be fitted to estimate the incidence rates of readmission per treatment group within 90 days after discharge. The result will be reported as a rate ratio (RR) and the Poisson model will be corrected for overdispersion if necessary.

To estimate the hazard ratio for the first readmission, again with starting time point at discharge, a Cox-model will be used.

The Fagerström test results, the quality of life (SF-36 Index) and the smoking status (cigarettes smoked per days) will be measured repeatedly over time from baseline, and for each outcome, mixed-effects models will be used to estimate the treatment effect and also to quantify the time*treatment interaction. The result of NicAlert Saliva test between treatment groups will be evaluated by applying chi-square test.

Cost-effectiveness analyses will be performed from a health care institution as well as a population public health perspective. First, hospital costs in both arms with and without costs of the tobacco treatment specialist (TTS) consultation per session, and nicotine replacement products will be compared as averages per patient across treatment groups. Second, the incremental cost per additional quit and incremental cost per additional life year saved of the preoperative institutional smoking cessation program compared to usual care will be calculated. Utilities will be calculated based on quality-of-life measurements using the SF-36 questionnaire.

### Interim analyses {21b}

A blinded restricted re-evaluation of the standard deviation assumed for the sample size calculation will be performed after an information rate (IR) of 40% is reached, meaning that 40% of the patients have completed the 90-day follow-up assessment of the primary outcome CCI. The estimation of the standard deviation will be performed on the combined set of patients of the intervention and control group, and therefore unblinding is not necessary. If the standard deviation at interim is larger than anticipated, an increase of the sample size will be discussed. No reduction of the sample size is planned. No statistical reasons for stopping the trial at interim have been specified. The trial may be stopped for other reasons, including slower recruitment than anticipated.

### Methods for additional analyses (e.g. subgroup analyses) {20b}

This is reported in the statistical analysis plan.

### Methods in analysis to handle protocol non-adherence and any statistical methods to handle missing data {20c}

Participants whose surgery is cancelled or takes place more than 4 months after randomization, do not attend for surgery or withdraw their consent before surgery or during the hospital stay will not be included in the intention to treat population. To ensure an adequate number of participants in the study, participants without surgery within 4 months after randomization and participants who withdraw their consent before surgery or during the hospital stay will be replaced by recruitment of new subjects. Patients included in the study but with the rescheduled date of surgery and <4 weeks between randomisation and surgery will not be excluded. Other missing data and postoperative loss of follow-up after three months will be addressed with multiple imputation in the primary analysis.

### Plans to give access to the full protocol, participant level-data and statistical code {31c}

The statistical analysis plan was submitted as Additional file 3 to the submission. The statistical code will be published as supplementary material, together with the main results of the study.

## Oversight and monitoring

### Composition of the coordinating centre and trial steering committee {5d}

The principal investigator will meet every Tuesday morning with the clinical nurse, and advanced nurse practitioner to discuss day-to-day support for the trial. The Trial Steering Committee consists of the principal investigator and the statistical head will meet regularly every 3 months.

### Composition of the data monitoring committee, its role and reporting structure {21a}

The data monitoring committee (DMC) will reassess the data in a blinded fashion as described to check the variability to reassess the sample size calculation.

### Adverse event reporting and harms {22}

An *adverse event (AE)* is any untoward medical occurrence in a patient or a clinical investigation subject which does not necessarily have a causal relationship with the trial procedure. An AE can therefore be any unfavourable or unintended finding, symptom, or disease temporally associated with a trial procedure, whether or not related to it.

A *serious adverse event (SAE)* (ClinO, Art. 63 [24]) is any untoward medical occurrence that

- Results in death or is life-threatening,
- Requires in-patient hospitalisation or prolongation of existing hospitalisation,
- Results in persistent or significant disability or incapacity, or
- Causes a congenital anomaly or birth defect

As the causal relationship between the events and the intervention can be ruled out, any event assessment regarding causality and severity will not be performed.

Both Investigator and Sponsor-Investigator make a severity assessment of the event as mild, moderate or severe. Mild means the complication is tolerable, moderate means it interferes with daily activities and severe means it renders daily activities impossible.

#### Reporting of SAEs (see ClinO, Art. 63)

The primary outcome and events on which the sample size is based represent postoperative complications which include SAEs. All SAEs within 90 days after surgery will be documented and analysed in this trial. As the origin of the SAEs is not intervention related, the SAEs are not actively reported to the EC in the applicable time limit of 15 days but are summarised in the annual safety report (ASR).

#### Notification of safety and protective measures (see ClinO, Art 62, b)

If immediate safety and protective measures must be taken during the conduct of the study, the investigator notifies the Ethics committee of these measures, and of the circumstances necessitating them, within 7 days.

### Frequency and plans for auditing trial conduct {23}

An annual safety report (ASR/DSUR) is submitted to the local Ethics Committee by the Investigator (ClinO, Art. 43 Abs) and will include postoperative complications described by the CCI within two unblinded safety analyses after 50 and 100 patients.

### Plans for communicating important protocol amendments to relevant parties (e.g. trial participants, ethical committees) {25}

Substantial changes to the study setup and study organization, the protocol and relevant study documents are submitted to the Ethics Committee for approval before implementation. Under emergency circumstances, deviations from the protocol to protect the rights, safety and well-being of human subjects may proceed without prior approval of the Ethics Committee. Such deviations shall be documented and reported to the Ethics Committee as soon as possible.

Substantial amendments are changes that affect the safety, health, rights and obligations of participants, changes in the protocol that affect study objectives or central research topic, changes of study site or of study leader and sponsor (ClinO, Art. 29).

A list of all non-substantial amendments will be submitted to the competent EC together with the ASR.

### Dissemination plans {31a}

Publications in peer-reviewed journals are planned.

## Discussion

The dedicated smoking cessation intervention is expected to cause sustained abstinence from smoking in a large percentage of the patients. The hypothesis is that the preoperative smoking cessation program improves outcomes in smokers undergoing surgery. The study will reveal if the expectations on successful smoking cessation rates are reasonable.

To date, only two RCTs have investigated the effects of an intensive preoperative smoking cessation program on postoperative complications [5, 6]. Even though the preoperative period is considered as a window of opportunity for cessation counselling randomized controlled evidence regarding its efficacy is still lacking. A systematic Cochrane Review showed evidence for increased short-term smoking abstinence in patients undergoing preoperative cessation counselling, whereas the effects on long-term abstinence and postoperative morbidity remained unclear [7].

In contrast to these previous studies, postoperative morbidity will be assessed using the CCI over a 90-day period. The CCI has been proven to be a strong endpoint, as it reflects the overall burden of the postoperative course in a single patient and thus allows for smaller sample sizes compared with traditional morbidity endpoints.

In line with most RCTs regarding preoperative cessation counselling, it is likely that the major limitations of our trial will be the relatively short preoperative period and the intensity of cessation counselling. To overcome these barriers our trial will only enrol patients who are scheduled for surgery with a preoperative period of more than one month and an intensive cessation program consisting of weekly behavioural support and pharmacotherapy will be implemented in as many patients as possible.

### Trial status

Recruitment will be started in June 2022 with the completion of recruitment in 2025.

## Supplementary Information

**Additional file 1.** Complications.

**Additional file 2.** Procedures.

**Additional file 3.** Statistical analysis plan.

## Data Availability

CDF will have access to the final trial dataset. Statistical programming will be initiated upon finalization of the data base. The deidentified dataset will be provided to the trial statistician for analysis.

## Abbreviations

AE: Adverse event
ASA: American Society of Anaesthesiologists
ASR/DSUR: Annual Safety Report/Development Safety Report
BASEC: Business Administration System for Ethical Committees
CCI: Comprehensive Complication Index
CCM: Chronic Care Model
ClinO: Ordinance on Clinical Trials in Human Research (in German: KlinV, in French: OClin, in Italian: OSRUm)
CRF: Case Report Form
CTCAE: Common Terminology Criteria for Adverse Events
FAS: Full analysis set
eCRF: Electronic Case Report Form
FOPH: Federal Office of Public Health
GCP: Good Clinical Practice
HADS: Hospital anxiety and depression scale
HRA: Human Research Act (in German: HFG, in French: LRH, in Italian: LRUm)
ICH: International Conference on Harmonisation
ITT: Intention to treat
SAE: Serious adverse event
SF-36: 36-Item Short Form Survey
TTS: Tobacco treatment specialist
TTM: Transtheoretical Model

## References

[1] Swiss Federal Statistical Office. Swiss Health Survey 2017. https://www.bfs.admin.ch/bfs/en/home/news/whats-new.assetdetail.6426306.html (Accessed 12 May 2022).

[2] Christopher Bullen CH, Laugesen M, McRobbie H, Parag V, Williman J, Walker N (2013). Electronic cigarettes for smoking cessation: a randomised controlled trial. Lancet.

[3] Hajek P, Phillips-Waller A, Przulj D, Pesola F, Myers Smith K, Bisal N (2019). A Randomized Trial of E-Cigarettes versus Nicotine-Replacement Therapy. N Engl J Med.

[4] Lee SM, Landry J, Jones PM, Buhrmann O, Morley-Forster P (2013). The Effectiveness of a Perioperative Smoking Cessation Program: A Randomized Clinical Trial. Anesth Analg.

[5] Møller AM, Villebro N, Pedersen T, Tønnesen H (2002). Effect of preoperative smoking intervention on postoperative complications: a randomised clinical trial. Lancet..

[6] Lindström D, Sadr Azodi O, Wladis A, Tønnesen H, Linder S, Nåsell H (2008). Effects of a perioperative smoking cessation intervention on postoperative complications: a randomized trial. Ann Surg.

[7] Thomsen T, Villebro N, Møller AM. Interventions for preoperative smoking cessation. Cochrane Database Syst Rev. 2014;2014(3):CD002294. 10.1002/14651858.CD002294.pub4. 10.1002/14651858.CD002294.pub4 PMC7138216 24671929

[8] Cornuz J, Humair JP, Seematter L, Stoianov R, van Melle G, Stalder H (2002). Efficacy of resident training in smoking cessation: a randomized, controlled trial of a program based on application of behavioral theory and practice with standardized patients. Ann Intern Med.

[9] Slankamenac K, Graf R, Barkun J, Puhan MA, Clavien PA (2013). The comprehensive complication index: a novel continuous scale to measure surgical morbidity. Ann Surg.

[10] Copeland GP, Jones D, Walters M (2005). POSSUM: A scoring system for surgical audit. Br J Surg.

[11] Wagner EH, Austin BT, Von Korff M (1996). Improving outcomes in chronic illness. Manag Care Q.

[12] Wagner EH, Austin BT, Von Korff M (1996). Organizing care for patients with chronic illness. Milbank Q.

[13] Coleman K, Austin BT, Brach C, Wagner EH (2009). Evidence On The Chronic Care Model In The New Millennium. Health Aff (Millwood).

[14] Haas JS, Linder JA, Park ER, Gonzalez I, Rigotti NA, Klinger EV (2015). Proactive Tobacco Cessation Outreach to Smokers of Low Socioeconomic Status. JAMA Intern Med.

[15] Donze J, Ruffieux C, Cornuz J (2006). Determinants of smoking and cessation in older women. Age Ageing.

[16] Cornuz JJSI, Humair J-P (2015). Ärztliche Rauchstoppberatung. Die Dokumentation für die Praxis.

[17] Clavien PA, Barkun J, de Oliveira ML, Vauthey JN, Dindo D, Schulick RD (2009). The Clavien-Dindo Classification of Surgical Complications: Five-Year Experience. Ann Surg.

[18] Slankamenac K, Nederlof N, Pessaux P, de Jonge J, Wijnhoven BP, Breitenstein S (2014). The comprehensive complication index: a novel and more sensitive endpoint for assessing outcome and reducing sample size in randomized controlled trials. Ann Surg.

[19] Tate JC, Schmitz JM (1993). A proposed revision of the fagerstrom tolerance questionnaire. Addict Behav.

[20] Bullinger M, Kirchberger I, Ware J (1995). Der deutsche SF-36 Health Survey Übersetzung und psychometrische Testung eines krankheitsübergreifenden Instruments zur Erfassung der gesundheitsbezogenen Lebensqualität. J Public Health.

[21] Zigmond AS, Snaith RP (1983). The Hospital Anxiety and Depression Scale. Acta Psychiatr Scand.

[22] Charlson ME, Pompei P, Ales KL, MacKenzie CR (1987). A new method of classifying prognostic comorbidity in longitudinal studies: development and validation. J Chronic Dis.

[23] Saklad M (1941). GRADING OF PATIENTS FOR SURGICAL PROCEDURES. Anesthesiology..

[24] Ordinance on Clinical Trials in Human Research (ClinO) https://www.admin.ch/opc/de/classified-compilation/20121176/index.html (Accessed 12 May 2022).

