# Supplementary material for: Preoperative smoking cessation program in patients undergoing intermediate to high-risk surgery: a randomized, single-blinded, controlled, superiority trial
Source: Trials. 2022 Aug 29;23:717. doi: 10.1186/s13063-022-06628-8 (PMC9422094; doi:10.1186/s13063-022-06628-8)
Supplement: Supplementary file 1 — Additional file 1. Complications. [file 13063_2022_6628_MOESM1_ESM.docx]

| **CODE** | **TYPE OF COMPLICATION** | | **MANAGEMENT** | | **CDC GRADE** | |
| --- | --- | --- | --- | --- | --- | --- |
|  |  | |  | |  | |
| **1** | **Infectious disorders** | |  | |  | |
|  |  | |  | |  | |
| 100 | Fever (>38°C) | | Antipyretic medication | | I | |
| 111 | Sepsis of unknown origin | | Life support (one organ failure), ICU | | IVa | |
| 112 | Sepsis of unknown origin | | Life support (multiple organ failure), ICU | | IVb | |
| 121 | Abscess | | Antibiotics | | II | |
|  | Abscess | | Image-guided drainage | | IIIa | |
|  | Abscess | | Drainage by surgery | | IIIb | |
| 131 | Urinary tract infection | | Antibiotics | | II | |
| 132 | Pyelonephritis | | Antibiotics | | II | |
| 133 | Epididym/Orchitis | | Antibiotics | | II | |
| 134 | Nephritis | | Antibiotics | | II | |
| 141 | Pneumonia | | Antibiotics | | II | |
| 142 | Endocarditis | | Antibiotics | | II | |
| 143 | Mediastinitis | | Antibiotics | | II | |
| 151 | Diverticulitis | | Antibiotics | | II | |
| 152 | Cholecystitis | | Antibiotics | | II | |
|  | Cholecystitis | | Surgery | | IIIb | |
| 153 | Gastroenteritis | | Antibiotics | | II | |
| 154 | Proctitis | | Antibiotics | | II | |
| 155 | Clostridium difficile colitis | | Antibiotics | | II | |
| 156 | Diarrhoea (non-infectious) | | Hydration, antidiarrheals, electrolytes | | I | |
| 157 | Diarrhoea | | Antibiotics | | II | |
| 161 | Osteomyelitis | | Antibiotics | | II | |
| 171 | Other infections | | Antibiotics | | II | |
| 181 | Erysipelas | | Antibiotics | | II | |
|  |  | |  | |  | |
| **2** | **Wound and skin** | |  | |  | |
|  |  | |  | |  | |
| 211 | Superficial wound infection | | Antibiotics | | II | |
| 212 | Deep wound infection | | Evisceration | | IIIa / IIIb | |
| 221 | Wound dehiscence | | Observation or diagnostic evaluation only, reinforced adhesive skin closure | | I | |
|  | Wound dehiscence | | Evisceration, new VAC-therapy | | IIIa / IIIb | |
| 231 | Hematoma | | Observation or diagnostic evaluation only | | I | |
|  | Hematoma | | Image-guided drainage | | IIIa | |
|  | Hematoma | | Drainage by surgery | | IIIb | |
| 241 | Seroma | | Observation or diagnostic evaluation only | | I | |
|  | Seroma | | Image-guided drainage | | IIIa | |
|  | Seroma | | Drainage by surgery | | IIIb | |
| 251 | Lymphocele / Lymphedema | | Observation or diagnostic evaluation only | | I | |
|  | Lymphocele | | Image-guided drainage | | IIIa | |
|  | Lymphocele | | Drainage by surgery | | IIIb | |
| 261 | Pressure skin ulcer | | Antibiotics, Bandage management | | II | |
| 271 | Incisional hernia | | Surgery | | IIIb | |
| 272 | Compartment syndrome | | Surgery | | IIIb | |
|  |  | |  | |  | |
| **3** | **Haematological disorders** | |  | |  | |
|  |  | |  | |  | |
| 311 | Intraoperative bleeding | | Blood transfusion | | II | |
| 312 | Postoperative bleeding | | Blood transfusion, tranexamic acid | | II | |
|  | Postoperative bleeding | | Surgery | | IIIb | |
| 313 | Low haemoglobin (f < 12 g/dl, m < 13.6 g/dl) | | Conservative, iron or vitamin supplementation | | I | |
|  | Low haemoglobin (f < 12 g/dl, m < 13.6 g/dl) | | Blood transfusion | | II | |
| 321 | Superficial thrombosis/phlebitis | | Conservative | | I | |
| 322 | Deep vein thrombosis | | Anticoagulation, elastic compression | | II | |
| 323 | Thrombosis other (ZVK) | | Anticoagulation | | II | |
| 331 | Pulmonary embolus | | Observation or diagnostic evaluation only | | I | |
|  | Pulmonary embolus | | Anticoagulation | | II | |
|  | Pulmonary embolus | | ICU, Anticoagulation | | IVa | |
| 341 | AV-Fistula | | Coiling | | IIIa | |
| 351 | Vascular injury | | Antibiotics | | II | |
|  |  | |  | |  | |
| **4** | **Cardiac disorders** | |  | |  | |
|  |  | |  | |  | |
| 411 | Hypotension | | Observation | | I | |
|  | Hypotension | | Medical treatment | | II | |
| 412 | Hypertension | | Observation | | I | |
|  | Hypertension | | Antihypertensives | | II | |
| 413 | Sinus tachycardia | | Medical treatment | | II | |
| 421 | Arrhythmia | | Medical cardioversion, anticoagulation | | II | |
|  | Arrhythmia | | Pacemaker, cardioversion | | IIIb | |
| 431 | Acute myocardial infarction | | Coronary intervention | | IVa | |
| 432 | Cardiac arrest | | CPR | | IVa | |
| 433 | Hypovolemic shock | | Fluid expansion, blood transfusion, VA-drugs | | II | |
| 434 | Angina pectoris | | Conservative, clinical observation or diagnostic evaluation only | | I | |
| 444 | Congestive heart failure excerbation | | Pharmacolgical Treatment | | II | |
|  |  | |  | |  | |
| **5** | **Pulmonary disorders** | |  | |  | |
|  |  | |  | |  | |
| 511 | Atelectasis | | Observation | | I | |
|  | Atelectasis | | Respiratory physiotherapy, CPAP | | II | |
| 512 | Respiratory distress/dyspnoe | | Oxygen, physiotherapy | | I | |
|  | Respiratory distress/dyspnoe | | Inhalation with Atrovent/Ventolin | | II | |
| 513 | Lung edema | | Observation | | I | |
|  | Lung edema | | Medical treatment | | II | |
| 514 | Pleural effusion | | Observation | | I | |
|  | Pleural effusion | | Thoracentesis | | IIIa | |
|  | Pleural effusion | | Thoracic drainage | | IIIa / IIIb | |
| 521 | Pneumothorax | | Observation | | I | |
|  | Pneumothorax | | Thoracic drainage | | IIIa / IIIb | |
| 531 | Respiratory failure | | Re-intubation | | IVa | |
| 541 | Anastomotic lung leak | |  | |  | |
| 551 | COPD exacerbation | | Inhalation with Atrovent/Ventolin | | II | |
| 561 | Blockage of tracheal tube | | Inhalation with Atrovent/Ventolin | | II | |
|  | Blockage of tracheal tube | | tracheoscopy | | IIIa | |
| **6** | **Gastrointestinal disorders** | |  | |  | |
|  |  | |  | |  | |
| 611 | Nausea/Vomiting | | Antiemetics and i.v. fluid support | | I | |
| 612 | Constipation | | Specific treatment | | II | |
| 613 | Ileus | | Observation; cessation of oral intake and i.v. fluid support | | I | |
|  | Ileus | | Conservative treatment/parenteral nutrition, gastric tube | | II | |
|  | Ileus | | Surgery | | IIIb | |
| 621 | Bowel injury (rectum included) | | Surgery | | IIIb | |
| 622 | Duodenal injury (fistula) | | Surgery | | IIIb | |
| 623 | Bowel ischemia | | Surgery | | IIIb | |
| 631 | Upper gastrointestinal bleeding | | Conservative; clinical observation or diagnostic evaluation only | | I | |
|  | Upper gastrointestinal bleeding | | Endoscopic sclerosis | | IIIa | |
|  | Upper gastrointestinal bleeding | | Surgery | | IIIb | |
| 632 | Lower gastrointestinal bleeding | | Conservative; clinical observation or diagnostic evaluation only | | I | |
|  | Lower gastrointestinal bleeding | | Endoscopic sclerosis | | IIIa | |
|  | Lower gastrointestinal bleeding | | Surgery | | IIIb | |
| 633 | Gastric ulcer | | Medical treatment | | II | |
| 641 | Enteric anastomosis leakage | | Surgery | | IIIb | |
| 642 | Enteric fistula | | Surgery | | IIIb | |
| 643 | Entero-cutaneous fistula | | Surgery | | IIIb | |
| 644 | Necrosis conduit | | Surgery | | IIIb | |
| 645 | High-output Stoma (> 2l/d) | | Hydration, Medical treatment | | II | |
| 651 | Oesophageal stenosis | | Surgery (stent) | | IIIb | |
| 661 | Blocked gastric / jejunal tube | | Unclogging / change of gastric tube | | I | |
| 662 | Loosened / ripped suture of intestinal tube/drain | | New suture | | II | |
| 671 | Ascites | | Puncturing | | IIIa | |
|  |  | |  | |  | |
| **7** | **Urological disorders** | |  | |  | |
|  |  | |  | |  | |
| 711 | Incontinence | | Observation | | I | |
| 712 | Catheter malfunction due to clot | | Desobstruction/chance catheter | | I | |
| 713 | Acute urinary retention (incl. after catheter removal) | | (Replacement of) catheter | | I | |
| 714 | Loss of catheter | | Endoscopic reinsertion | | IIIa | |
| 715 | Double J stent encrustation | | Surgery, ESWL | | IIIb | |
| 715 | Dislocated ureter catheter | | Surgery | | IIIb | |
| 721 | Urinary leak | | Observation | | I | |
|  | Urinary leak | | Surgery | | IIIb | |
| 722 | Urinoma | | Observation | | I | |
|  | Urinoma | | Drainage by surgery | | IIIb | |
| 731 | Ureteroenteric anastomotic leak | | Conservative | | I | |
|  | Ureteroenteric anastomotic leak | | Endoscopy/nephrostomy | | IIIa | |
|  | Ureteroenteric anastomotic leak | | Surgery | | IIIb | |
| 732 | Conduit bleeding | | Surgery | | IIIa | |
| 733 | Urethral anastomosis leak | | Conservative | | I | |
|  | Urethral anastomosis leak | | Surgery | | IIIb | |
| 734 | Urostomy hernia | | Surgery | | IIIb | |
| 735 | Urostomy ischemia | | Surgery | | IIIb | |
| 741 | Transient hydronephrosis | | Observation | | I | |
| 742 | Persistent hydronephrosis | | Stent | | IIIa | |
| 743 | Acute renal insufficiency  ● Increase in serum creatinine by ≥0.3 mg/dL (≥26.5 micromol/L) within 48 hours, or  ●Increase in serum creatinine to ≥1.5 times baseline, which is known or presumed to have occurred within the prior seven days, or  ●Urine volume <0.5 mL/kg/hour for six hours | | Conservative treatment | | I | |
| 744 | Acute renal insufficiency | | Dialysis | | IVa | |
| 751 | Ureteral stenosis | | Ureteral stent | | IIIa | |
| 761 | Bacteriuria (>10^5^ cfu/ml, asymptomatic) | | Conservative, no antibiotics | | I | |
| 762 | Hematuria | | Conservative; clinical observation or diagnostic evaluation only | | I | |
|  |  | |  | |  | |
| **8** | **Neurological disorders** | |  | |  | |
|  |  | |  | |  | |
| 811 | Cerebrovascular accident/TIA | | Conservative | | I | |
|  | Cerebrovascular accident | | Thrombolysis, Anticoagulation, antiplatelets | | II | |
|  | Cerebrovascular accident | | Surgery | | IIIb | |
| 821 | Convulsion | | Medical treatment | | II | |
| 822 | Loss of consciousness/syncope | | Conservative | | I | |
| 823 | Vertigo | | Conservative | | I | |
| 824 | Vertigo | | Medical treatment | | II | |
| 831 | Mental confusion/altered mental status/Delirium | | Hydration, support, antipsychotics | | II | |
| 833 | Alcohol withdrawal syndrome | | Benzodiazepine | | II | |
| 832 | Depression | | Observation | | I | |
|  | Depression | | Medical treatment | | II | |
| 841 | Peripheral neuropathy | | Observation | | I | |
|  | Peripheral neuropathy | | Medical treatment | | II | |
|  | Peripheral neuropathy | | Surgery | | IIIb | |
|  |  | |  | |  | |
| **9** | **Gynaecologicaldisorders** | |  | |  | |
|  |  | |  | |  | |
| 911 | Rectovaginal fistula | | Surgery | | IIIb | |
|  |  | |  | |  | |
|  |  | |  | |  | |
|  |  | |  | |  | |
|  |  | |  | |  | |
|  |  | |  | |  | |
|  |  | |  | |  | |
|  |  | |  | |  | |
|  |  | |  | |  | |
|  |  | |  | |  | |
|  |  | |  | |  | |
| **10** | **Vascular disorders** | |  | |  | |
|  |  | |  | |  | |
| 1010 | Artery bypass obstruction | | PTA | | IIIa | |
|  | Artery bypass obstruction | | Open graft, Amputation | | IIIb | |
|  |  | |  | |  | |
|  |  | |  | |  | |
|  | **Others** | |  | |  | |
|  |  | |  | |  | |
| 11 | Foreign body intracorporal | | Surgery | | IIIb | |
| 21 | Acidosis | | Medical treatment, electrolytes | | I | |
| 22 | Hypokalemia | | Medical treatment | | I | |
| 23 | Dehydration | | i.v. fluid support | | I | |
| 31 | Edema | | Medical treatment | | I | |
| 41 | Dermatitis | | Ointment | | I | |
| 51 | Pain exacerbation | | Medical treatment | | I | |
| 61 | Readmission | | Analgetics, Hydration etc. | | II | |
|  |  | |  | |  | |
|  | |  | |  | |  |
|  | |  | |  | |  |
|  | |  | |  | |  |
|  | |  | |  | |  |
|  | |  | |  | |  |
|  | |  | |  | |  |
|  | |  | |  | |  |
|  | |  | |  | |  |
|  | |  | |  | |  |
|  | |  | |  | |  |
|  | |  | |  | |  |
|  | |  | |  | |  |
|  |  | |  | |  | |
|  |  | |  | |  | |
|  |  | |  | |  | |
|  |  | |  | |  | |
|  |  | |  | |  | |
|  |  | |  | |  | |
|  |  | |  | |  | |
|  |  | |  | |  | |
