# Supplementary material for: Preoperative smoking cessation program in patients undergoing intermediate to high-risk surgery: a randomized, single-blinded, controlled, superiority trial
Source: Trials. 2022 Aug 29;23:717. doi: 10.1186/s13063-022-06628-8 (PMC9422094; doi:10.1186/s13063-022-06628-8)
Supplement: Supplementary file 2 — Additional file 2. Procedures. [file 13063_2022_6628_MOESM2_ESM.docx]

| **Intermediate risk procedures** | **High risk procedures** |
| --- | --- |
| **Abdominal surgery** | |
| Any planned open, laparoscopic or robotic resections or biopsies of the following organs: small or large bowel, spleen or peritoneum and/or adjacent lymph nodes in the retroperitoneum, abdomen, pelvis or groins. | Esophagectomy, gastrectomy, hepatectomy, pancreatectomy, duodenectomy, abdominoperinoeal resections |
| **Gynaecology** | |
| Any planned open, laparoscopic or robotic resections or biopsies the gynecological organs ovaries, fallopian tube, uterus, cervix and/or adjacent lymph nodes in the retroperitoneum, abdomen, pelvis or groins. Excluded vulvectomies without adjunct procedures (e.g. groin node dissection, cystectomy, flap reconstruction). | **-** |
| **Thoracic surgery** | |
| Any planned open, laparoscopic or robotic resections or biopsies within the chest and/or adjacent lymph nodes in the retroperitoneum or chest. Excluded are bronchoscopy procedures, pleurX like catheter implantations or pleurodesis. | **-** |
| **Urology** | |
| Any planned open, laparoscopic or robotic resections or biopsies of the following organs: adrenal, kidney, ureter, bladder, urethra and/or adjacent lymph nodes in the retroperitoneum, abdomen, pelvis or groins. Excluded are men undergoing prostatectomy, endourologic procedures, testicular or penile surgery without lymph node surgery. | Cystectomy |
| **Head neck** | |
| Any planned open, laparoscopic or robotic resections of head and neck surgeries. Excluded are endoscopic procedures and biopsies. |  |

Adapted from [1]

[1] Copeland G, Jones D, Walters M. POSSUM: a scoring system for surgical audit. British Journal of Surgery. 1991;78:355-60.
