# Supplementary material for: Preoperative smoking cessation program in patients undergoing intermediate to high-risk surgery: a randomized, single-blinded, controlled, superiority trial
Source: Trials. 2022 Aug 29;23:717. doi: 10.1186/s13063-022-06628-8 (PMC9422094; doi:10.1186/s13063-022-06628-8)
Supplement: Supplementary file 3 — Additional file 3. Statistical analysis plan. [file 13063_2022_6628_MOESM3_ESM.pdf]

Epidemiology, Biostatistics
and Prevention Institute,
Biostatistics Department

www.biostat.uzh.ch

Preoperative smoking cessation program in patients undergoing intermediate to high-risk surgery: a randomized, single-blinded, controlled trial

Statistical Analysis Plan

MSc Beatrice Cortonesi (*b.cortonesi@icloud.com*)
Prof. Dr. Ulrike Held (*ulrike.held@uzh.ch*)
Research Team: Milo A. Puhan, Christian D Frankhauser

Version 1.0 of April 15, 2022

DISCLAIMER

This report was written by the Research Methods Consulting Unit of the University of Zurich, Epidemiology, Biostatistics and Prevention Institute, Biostatistics Department (EBPI). Any citation of results or comments (or parts thereof) given in this report in any format (including: paper, presentation, abstract, grant application) or the naming of EBPI or persons of the EBPI requires the explicit approval of the senior statistician (Prof. Dr. Ulrike Held). This is especially true for citations in the "acknowledgments".

Contents

1 Introduction **1**
1.1 Background and rationale 1
1.2 Objectives 2

2 Trial Methods **2**
2.1 Trial design 2
2.2 Randomization 2
2.3 Sample size 2
2.4 Framework 2
2.5 Interim analysis and stopping guidance 2
2.6 Timing of final analysis 3
2.7 Timing of outcome assessments 3

3 Statistical Principles **3**
3.1 Confidence intervals and P values 3
3.2 Adherence and Protocol deviations 4
3.2.1 Definition of adherence to the intervention and how this is assessed 4
3.2.2 Description of how adherence to the intervention will be presented 4
3.2.3 Definition of protocol deviation for the trial 4
3.2.4 Description of which protocol deviations will be summarised 4
3.3 Analysis populations 4

4 Trial Population 5
4.1 Screening data 5
4.2 Eligibility 5
4.3 Completion of the study 6
4.4 Withdrawal/ Follow-up 6
4.4.1 Level of withdrawal 6
4.4.2 Timing of withdrawal 7
4.4.3 Reasons for withdrawal 7
4.5 Baseline patient characteristics 7

5 Analysis 9
5.1 Outcome definitions 9
5.2 Analysis methods 11
5.2.1 Cost-effectiveness analyses 12
5.2.2 Subgroup Analysis 14
5.3 Missing data 15
5.4 Additional analyses 15
5.5 Harms 18
5.6 Statistical software and programming 19

6 Additional remark 19

7 References 20

1 Introduction

1.1 Background and rationale

One quarter of the Swiss population smokes daily [1]. A unique opportunity to initiate smoking cessation focuses on smokers scheduled for surgery. These patients are not only highly motivated to quit smoking [2] but also likely to benefit from a reduction in postoperative complications of 40% which may translate into a decrease of costs by 10% [3-5]. A systematic Cochrane review enrolling 13 trials [5] concluded that preoperative smoking interventions increase short-term smoking cessation and reduces postoperative complications. However, previous studies only reported postoperative complications up to a 30-day postoperative period and complications were not graded by severity. Additionally, most studies were performed in orthopedic departments and therefore limiting the generalizability of its results. We therefore aim to analyse the impact of a preoperative high-intensity smoking cessation intervention on surgical complications up to a 90-day postoperative period in patients of various surgical disciplines. Further advantages of this study represents the throughout coding and definition of postoperative complication and institutional approach. In this trial we will report complications according to the Comprehensive Complication Index (CCI®) [6], which represents the most widely used and accepted measure of post-operative complications. Thereby, we improve reporting of postoperative morbidity since complications are graded by severity and the cumulative burden from any combination of complications is described in a single patient.

1.2 Objectives

The hypothesis is that a preoperative smoking cessation program improves outcomes in smokers undergoing surgery. The primary objective is to compare complications between patients with an institutional multifaceted smoking cessation intervention compared to patients in the advice only group (control group) within a 90-day postoperative period. Secondary objectives include comparison of smoking abstinence, quality of life, mental health, length of hospital stay, costs of care and difference in hospital reimbursement between the two groups.

2 Trial Methods

2.1 Trial design

This investigation is a monocentric, randomized, single blinded, controlled, superiority trial involving patients undergoing intermediate and high-risk surgery [7] at the cantonal hospital of Lucerne. Patients will be randomised in a 1:1 ratio to either the intervention or control group.

2.2 Randomization

Before randomisation patients will be pre-stratified for age ($\leq$ 60 years, $>$ 60 years) and procedure (intermediate versus high-risk procedures). Both factors have been identified in a previous retrospective cohort study at our institution as risk factors for complications. Minimization is an efficient way to control for confounding in small to moderately sized trials and will be used here.

2.3 Sample size

Based on a previous Cochrane review with meta-analysis [7], it is assumed that preoperative smoking cessation decreases the binary outcome postoperative complications with a relative risk of 0.42. The mean postoperative CCI as continuous measure in a retrospective chart review in a cohort without smoking cessation at the hospital of Lucerne was 13 and we therefore assume a CCI of 5.5 in the intervention group. The assumed standard deviation for the sample size calculation was 20. With anticipated 80% power, two-sided significance level α of 5%, a sample size of 226 patients is planned. With additional 10% dropout our aim is to enrol 251 patients.

2.4 Framework

The null hypothesis is that preoperative smoking cessation has no impact on postoperative complications occurring within 90 days after intermediate to high-risk surgery. The alternative hypothesis would be that there is a difference in postoperative complications between patients undergoing preoperative smoking cessation compared to the control group.

2.5 Interim analysis and stopping guidance

A blinded restricted re-evaluation of the standard deviation assumed for the sample size calculation will be performed after an information rate (IR) of 40% is reached, meaning that 40% of the patients have completed the 90-day follow-up assessment of the primary outcome CCI. The estimation of the standard deviation will be performed on the combined set of patients of treatment and control group, and therefore unblinding is not necessary. If the standard deviation at interim is larger than anticipated, an increase of the sample size will be discussed. No reduction of the sample size is

planned. No statistical reasons for stopping the trial early have been specified. The trial may be stopped for other reasons, including slower recruitment than anticipated.

2.6 Timing of final analysis

Data used for the primary outcome analysis will be ready after all the patients have completed the 90 days post-operative period. Secondary analyses will be carried out at the end of the 12-month follow-up. The results on the primary outcome will be reported first. Secondary analyses will be reported later.

2.7 Timing of outcome assessments

The schedule of assessments is shown in the image below.

**Time**	**- 1 week**	**0**		**2–3 weeks**	**> 4 weeks ≤ 4 months**		**3 month (+- 10 days)**	**6 month (+- 10 days)**	**12 (+- 2 months)**
	Information call	Screening	Contact	1st Visit	Admission	Discharge	Follow -up	Follow -up	Follow -up
**Recruitment**	+								
**Oral and written patient information**	+								
**Written consent**		+							
**Inclusion-/ exclusion criteria**		+							
**Questionnaire (nicotine dependency, quality of life)**			+				+	+	+
**Questionnaire (mental health, physical activity, alcohol cunsumption)**			+						
**Randomisation**		+							
**Safety**		+							
**Medical history**		+							
**Participant characteristics**		+							
**Intervention**				+					
**Surgery**					+				
**Complication Assesment**						+	+	+	+
**NicAlert test**									+

3 Statistical Principles

3.1 Confidence intervals and P values

All applicable statistical tests will be performed using a 2-sided 5% significance level and all confidence intervals presented will be 95% and two-sided.

3.2 Adherence and Protocol deviations

3.2.1 Definition of adherence to the intervention and how this is assessed

Adherence to the intervention is defined as the participation in at least the first smoking cessation counselling and it will be assesed by checking the date of the first smoking cessation counselling.

3.2.2 Description of how adherence to the intervention will be presented

Collected Variables

- Date of the initial consultation (yyyy-mm-dd) [Questionary 4a]

Derived Variables

- Whether or not a patient participated in at least the first smoking cessation consultation (Yes/No)

Adherence to the intervention will be summarised for the intervention group as follows:

Table 1 *Frequency tabulation of patients who participated and who did not participate in at least the first smoking cessation consultation.*

3.2.3 Definition of protocol deviation for the trial

Protocol deviation is defined as participant not scheduled for first smoking cessation intervention or participant does not show up at intervention.

3.2.4 Description of which protocol deviations will be summarised

Derived Variables

- Whether a patient has not scheduled for first smoking cessation intervention
- Whether a patient does not show up at intervention

Protocol violations will be summarised as follows:

Table 2 *The frequency of patients with protocol deviation will be presented.*

3.3 Analysis populations

The following populations will be considered:

- **Full Analysis Set (FAS)**: All randomised patients, according to the intention to treat (ITT) principle who had the surgery within 4 months from randomization.
- **Per Protocol Analysis Set**: All patients who completed the study without any protocol violation
- **Safety Analysis Set**: All randomised patients, according to the intention to treat (ITT).

4 Trial Population

4.1 Screening data

Collected variables

- Patient identification (tumor board, surgery list or referral) [Questionary 1]
- Patient enrolled in study (Yes, Not meeting inclusion criteria, Eligible but not interested, Eligibility unclear, Eligible but consent missing) [Questionary 1]
- Reason for premature end of study (lost to follow-up, withdrawal of consent, death, surgery not done) [Questionary 11]

Analysis Sets to be used: All screened patients

Representativeness of trial sample will be described as follows:

Table 3 *Frequency of enrolled patients, frequency of patients who do not meet inclusion criteria, frequency of eligible patients but not interested, frequency of patients with unclear eligibility, frequency of eligible patients but consent missing will be presented.*

Table 4 *Frequency of patients who did not had surgery within 4 months from randomization and frequency of replaced patients will be presented.*

In addition to the table described above, a figure will present representativeness of trial sample in the form of a flowchart:

Figure 1 *The number of screened patients, the number of screened patients identified by tumor board, surgery list or referral, the number of recruited patients and not recruited along with the reason for non recruitment will be presented in the form of a flowchart.*

4.2 Eligibility

Collected variables

Inclusion:

- Surgical treatment planned (Yes/No) [Questionary 1]
- Intermediate risk or high risk surgery according to protocol (Yes/No) [Questionary 1]
- Surgery more than 4 weeks and $\leq$ 4 months between tumor board/listing and surgery (Yes/No) [Questionary 1]
- More than 1 cigarette, cigar or pipe per day (Yes/No) [Questionary 1]
- Patient date of birth (yyyy-mm-dd) [Questionary 1]
- Written informed consent (Yes/No) [Questionary 1]

Exclusion:

- Department of surgery (abdominal surgery, thoracic surgery, urology, gynecology, vascular

- surgery, head and neck surgery, other) [Questionary 1]
- Patient does not consume illegal drugs (Yes/No) [Questionary 1]
- Patient does not suffer from alcohol related disorders (Yes/No) [Questionary 1]
- Native tongue (German/Does not speak german fluently) [Questionary 1]
- Patient does not suffer from dementia or other mental illnesses (Yes/No) [Questionary 1]

Derived variables A variable saying Yes if the patient is over 18 years old and No otherwise.

Analysis Sets to be used: All screened patients

Eligibility criteria will be summarised as follows:

Table 5 *Total number of screened patients, number of screened patients who meet and not meet inclusion/exclusion criteria will be presented.*

4.3 Completion of the study

Collected variables

- Did participant complete the study (Yes/No) [Questionary 11]
- Reason for premature end of study (lost to follow-up, withdrawal of consent, death, surgery not done) [Questionary 11]

Analysis Sets to be used: All randomised patients

Patients disposition will be summarised by treatment group as follows:

Table 6 *Number of randomised patients, number of patients in the Full Analysis Set (FAS), in the Per Protocol Analysis Set and in the Safety Analysis Set will be presented both overall and by treatment group*

In addition, a figure will present patients disposition in the form of a flowchart:

Figure 2 *The total number of randomised patients,the number of randomised patients per treatment group, the number of patients who completed the study and who did not complet the study with reasons by treatment group will be presented in the form of a flowchart.*

4.4 Withdrawal/ Follow-up

4.4.1 Level of withdrawal

Collected variables

- Date of withdrawal of consent (yyyy-mm-dd) [Questionary 11]
- Date of admission for surgery (yyyy-mm-dd) [Questionary 1]

Derived variables Whether the patients withdraw consent in preoperative or within a 90-day postoperative period or after the 90-day postoperative period.

Analysis Sets to be used: All randomised patients

Level of consent withdrawal will be summarised as follow:

Table 7 *Number of patients who withdraw consent with total data elimination, number of patient who withdraw consent but data kept will be presented by treatment group.*

4.4.2 Timing of withdrawal

Collected variables

- Date of answering questionnaire (yyyy-mm-dd) [Questionary 2]
- Date of answering questionnaire (yyyy-mm-dd) [Questionary 5]
- Date of answering questionnaire (yyyy-mm-dd) [Questionary 7(3 , 6 or 12 month)]
- Reason for premature end of study (lost to follow-up, withdrawal of consent, death, surgery not done) [Questionary 11]

Analysis Sets to be used: All randomised patients

Timing of withdrawal will be summarised by treatment group as follows:

Figure 3 *At 3 weeks before surgery, at 1 week before surgery and at 3, 6 and 12 months after surgery, the number of randomised patients who continue the study, the number of drop-out patients with reason will be presented in the form of a flowchart per treatment group.*

4.4.3 Reasons for withdrawal

Collected variables

- Reason for premature end of study (lost to follow-up, withdrawal of consent, death, surgery not done) [Questionary 11]

Analysis Sets to be used: All randomised patients

Reason for premature end of study will be summarised by treatment group as follows:

Table 8 *For premature discontinuation reason, a default frequency tabulation will be provided.*

4.5 Baseline patient characteristics

Collected variables

- Patient date of birth (yyyy-mm-dd) [Questionary 1]
- Intermediate or high-risk procedure (Intermediate risk, high risk) [Questionary 1]
- Patient gender (Male, Female) [Questionary 1]

- Patient identification (tumor board, surgery list or referral) [Questionary 1]
- Department of surgery (abdominal surgery, thoracic surgery, urology, gynecology, vascular surgery, head and neck surgery, other) [Questionary 1]
- More than 1 cigarette, cigar or pipe per day (Yes, No) [Questionary 2]
- Smoking products (Zigaretten, E-Zigaretten, Tabakpfeiffe, Zigarren, Andere) [Questionary 2]
- Cigarettes smoked per day in the past few days (Numeric) [Questionary 2]
- Age at which patient started smoking (Numeric) [Questionary 2]
- Total years of smoking at least 1 cigarette, cigar or pipe per day (Numeric) [Questionary 2]
- Average cigarettes smoked per day overall years of smoking (Numeric) [Questionary 2]
- Packyears (Numeric) [Questionary 2]
- Cigarettes smoked per day in year before surgery (Numeric) [Questionary 2]
- Smoking cessation attempts before study (Yes/No) [Questionary 2]
- Number of previous smoking cessation attempts (Numeric) [Questionary 2]
- Number of previous smoking cessation attempts in the last year (Numeric) [Questionary 2]
- Year of last smoking cessation attempt before surgery (yyyy) [Questionary 2]
- Longest smoking free period (days) [Questionary 2]
- Pharmaceutical treatment in previous smoking cessation attempt (Yes/No) [Questionary 2]
- Type of pharmaceutical treatment in previous smoking cessation attempt (Nikotinersatz (z.B. Nikotinpflaster, Nikotinkaugummi), Champix (varenicline), Zyban (bupropion), E-Zigarette, Andere) [Questionary 2]
- Smoking persons in patients home environment (Yes, No) [Questionary 2]
- Smoking persons in patients work environment (Yes, No) [Questionary 2]
- Reasons to quit smoking (Gesundheitsrisiko, Symptome, Abhängigkeit, Sozialer Druck, Kosten, Anderes) [Questionary 2]
- Other reason to quit smoking (string) [Questionary 2]
- Motivation scale to quit smoking (0, 1, . . . , 10) [Questionary 2]
- Barriers to quit smoking (Entzugssymptome, Verlust des Vergnügens, Gewichtszunahme, Stress, Rückfallsangst, Anderes) [Questionary 2]
- Other barriers to quit smoking (string) [Questionary 2]
- Confidence scale in successful smoking cessation (1, 2, . . . , 10) [Questionary 2]
- Quality of life (SF-36 Index) total score (Numeric) [Questionary 2]

- Morbility (Charlson Comorbidity Index) total score (Numeric) [Questionary 3]
- Result of Fagerstroem test for nicotine dependence (1, 2, . . . , 10) [Questionary 2]
- American Society of Anaesthesiologists (ASA) classification (1, 2, 3, 4, 5, 6) [Questionary 3]
- Obesity as cardiovascular risk factor (yes, unknown, no) [Questionary 3]
- Arterial hypertension as cardiovascular risk factor (yes, unknown, no) [Questionary 3]
- Dyslipidemia as cardiovascular risk factor (yes, unknown, no) [Questionary 3]
- Depression (yes, unknown, no) [Questionary 3]
- Total number of medical diagnoses (Numeric) [Questionary 3]
- Stage of transtheoretical model (TTM) for smoking cessation (precontemplation, contemplation, preparation) [Questionary 2]

Derived variables
Patient age

Analysis Sets to be used: Full Analysis Set (FAS)

Baseline patient characteristics will be summarised by treatment group as follows:

Figure 4 *Box plots will show the distribution of "Patient age", "Cigarettes smoked per day in the past few days", "Age at which patient started smoking", "Total years of smoking at least 1 cigarette, cigar or pipe per day", "Average cigarettes smoked per day overall years of smoking", "Packyears", "Cigarettes smoked per day in year before surgery", "Number of previous smoking cessation attempts", "Number of previous smoking cessation attempts in the last year", "Longest smoking free period", "Quality of life (SF-36 Index)", "Morbility (Charlson Comorbidity Index)", "Result of Fagerstroem test for nicotine dependence", " American Society of Anaesthesiologists (ASA) classification" and "Total number of medical diagnoses " and they will be presented both overall and separately per treatment group.*

Table 9 *Frequency tabulations for all the other collected variable will be presented both overall and separately per treatment group.*

5 Analysis

5.1 Outcome definitions

The primary endpoint of the study is the Comprehensive Complication Index (CCI®) within 90 days of surgery.

Collected variables

- Patient date of birth (yyyy-mm-dd) [Questionary 1]
- Intermediate or high-risk procedure (Intermediate risk, high risk) [Questionary 1]
- Comprehensive Complication Index (Numeric) [Questionary 6]

Derived variables

- Whether the patient is $\leq$ 60 or >60 years old

Secondary endpoints are length and costs of hospital stay, readmission rates for inpatient hospital stay, smoking abstinence or nicotine reduction, nicotine dependence, mental health, quality of life and unplanned postoperative intermediate care or intensive care unit admission cost. Secondary endpoints will be recorded up to a 12-month postoperative follow-up period and will be compared between the intervention and control group.

Collected variables

- Result of Fagerström test (Numeric) [Questionary 2, 7 (3 , 6 or 12 month)]
- Mental Health HADS total score (0, 1, . . . , 42) [Questionary 5]
- Quality of life (SF-36 Index) total score (Numeric) [Questionary 2, 7 (3 , 6 or 12 month)]

Smoking abstinence or nicotine reduction:

- Cigarettes smoked per day in the past few days (number cigarettes/days) [Questionary 2]
- Cigarettes smoked per day in the last week (number cigarettes/days) [Questionary 5]
- Cigarettes smoked per day after surgery (number cigarettes/days) [Questionary 2, 7 (3 , 6 or 12 month)]

Length and cost of hospital stay:

- Date of admission for surgery (yyyy-mm-dd) [Questionary 9]
- Date of discharge from hospital after surgery (yyyy-mm-dd) [Questionary 9]
- Hospital costs for planned surgery (Numeric - CHF) [Questionary 9]

Readmission Rates:

- Date of first unplanned readmission after surgery (yyyy-mm-dd) [Questionary 6]
- Date of second unplanned readmission after surgery (yyyy-mm-dd) [Questionary 6]

Unplanned postoperative intermediate care or intensive care unit admission:

- Hospital costs for unplanned readmission (Numeric - CHF) [Questionary 9]
- Costs for unplanned outpatient visits (Numeric - CHF) [Questionary 9]

Derived variables

- Length of hospital stay = days between the date of admission for surgery and the date of discharge from hospital after surgery
- Cost of unplanned postoperative care = hospital costs for unplanned readmission + costs for unplanned outpatient visits
- A variable indicating days between the date of discharge from hospital after surgery and the date of first unplanned readmission after surgery (event of interest) or the censored time date or the death date before readmission (competing event) and another variable indicating if the event of interested happened.

5.2 Analysis methods

Primary outcome CCI® will be addressed with a multiple linear model, including the randomized treatment group and the minimization variables (age $\leq$ 60 or >60 and intermediate or high-risk surgery) as independent variables. If after normality check using the qq-plot the residual distribution is skewed a transformation of the CCI will be necessary. To evaluate the between-group difference in CCI of the smoking cessation intervention versus control group, adjusted for the above defined confounders, the Wald test to check the significance of individual regression coefficients will be used.

Analysis Sets to be used: Full Analysis Set (FAS) and Per Protocol Analysis Set

The treatment effects for the primary outcome will be presented as follows:

Table 10 *The estimated effect of the treatment group, choosing controls as the reference group, along with the 95% confidence interval and the associated p-value from Wald test will be presented.*

For each secondary outcome such as the length and cost of hospital stay, the mental health HAD Scale and the cost of unplanned postoperative care a simple linear regression model with the randomized treatment group as independent variable will be used to estimate the differences between treatment groups. To evaluate the between-group difference in length and cost of hospital stay, in the mental health HAD Scale and in the cost of unplanned postoperative care of the smoking cessation intervention versus control group the Wald test to check the significance of individual regression coefficients will be used. In addition to that, the estimated means and confidence intervals for each secondary outcome will be presented per treatment group. Normality will be checked using qq-plot and, if necessary, appropriate transformations of the dependent variables will be made.
For first readmission rates, the date of discharge from hospital after surgery will be chosen as the starting observational time. The Poisson model, considering readmissions collectively, having the randomised treatment group as independent variable, will be used to estimate the Incidence Rates of readmission per treatment group, the Rate Ratio (RR) and the associated Wald confidence interval. The Poisson model will be corrected for overdispersion if necessary. To evaluate the statistical significance of the treatment effect the Wald Chi-Square test will be applied. In addition, considering the first readmission as the event of interested a Cox-model will be used to estimate the Hazard Ratio (HR) between treatment groups and the associated P-value form Chi-square Test to evaluate a possible difference between the treatment groups will be presented. If competing events like death before readmission are present, the Fine-Gray model to estimate the treatment effect on the subdistribution hazard function will be used. For second readmission rates, the absolute risk of second readmission will be calculated in the population of patients that had a first readmission and the Relative Risk (RR) or the Absolute risk reduction (ARR) between the treatment group will be shown. Confidence intevals of Relative Risk will be provided using the normal approximation method.
As the Fagerström test results, the quality of life (SF-36 Index) and the cigarettes smoked per days are measured repeatedly over time, for each one mixed-effects models will be used to see if there is a time*treatment interaction. To test the hypothesis of no time*treatment interaction the type III anova tables for fixed-effect terms with Satterthwaite and Kenward-Roger methods for denominator degrees of freedom for F-tests will be provided.

Analysis Sets to be used: Full Analysis Set (FAS)

Differences in treatment groups for secondary outcome will be presented as follows:

Table 11 *Estimated means by treatment groups and estimated treatment effects along with their confidence intervals for length of hospital stay, cost of hospital stay, mental health HAD Scale results and cost of unplanned postoperative care and the associated P-values from Wald Test.*

Table 12 *The Incidence Rates of first readmission per treatment group and the Rate Ratios (RR) along with the 95% confidence intervals and associated P-values will be presented.*

Table 13 *The estimated Hazard Ratio of first readmission between treatment groups and the associated P-value will be presented.*

Table 14 *The Absolute Risk (AR) of second readmission per treatment group and the Relative Risk (RR) or Absolute risk reduction (ARR) of second readmission between the treatment group will be presented along with confidence intervals.*

Table 15 *P-values associated with treatment, time and time*treatment from F-test for Fagerström test results, the quality of life (SF-36) Index and cigarettes smoked per days and the estimated fixed effects will be presented.*

Figure 5 *Spaghetti plot over time for Fagerström test result by treatment group.*

Figure 6 *Spaghetti plot over time for quality of life (SF-36) Index by treatment group.*

Figure 7 *Spaghetti plot over time for cigarettes smoked per days by treatment group.*

Figure 8 *Box-plot of Fagerström test result distribution at each time point by treatment group.*

Figure 9 *Box-plot of quality of life (SF-36) Index distribution at each time point by treatment group.*

Figure 10 *Box-plot of cigarettes smoked per days distribution at each time point by treatment group.*

5.2.1 Cost-effectiveness analyses

Collected variables

- Hospital costs for planned surgery (Numeric - CHF) [Questionary 9]
- Hospital costs for unplanned readmission (Numeric - CHF) [Questionary 9]
- Costs for unplanned outpatient visits (Numeric - CHF) [Questionary 9]
- Tobacco treatment specialist (TTS) cost (Numeric - CHF)
- Nicotine replacement products cost (Numeric - CHF)
- Last date of smoking cessation [Questionary 5]

Derived variables

- Total hospital costs per patient = hospital costs for planned surgery + hospital costs for unplanned readmission + costs for unplanned outpatient visits + tobacco treatment specialist (TTS) cost + nicotine replacement products cost

- Total hospital costs per patient without costs of the tobacco treatment specialist (TTS) and nicotine replacement products = hospital costs for planned surgery + hospital costs for unplanned readmission + costs for unplanned outpatient visits
- A variable that tells whether a patients has successfully quit smoking before surgery
- A variable that tells whether a patients has died after surgery
- Incremental cost-effectiveness ratio for additional success in quitting smoking:

$$ICER = \frac{C_{treatment} - C_{control}}{E_{quit\ with\ treatment} - E_{quit\ with\ control}} \quad (1)$$

  where $C_{treatment}$ is the average cost of smoking cessation treatment per patient, $C_{control}$ is the cost of control treatment. Costs of the intervention will be calculated based on unit costs of counselling meetings and nicotine replacement therapy. $E_{quit\ with\ treatment}$ and $E_{quit\ with\ control}$ are the percentage of patients who have successfully quit smoking before surgery in the treatment group and in the control group respectively

- Incremental cost-effectiveness ratio for additional life year saved:

$$ICER = \frac{C_{treatment} - C_{control}}{E_{alive\ with\ treatment} - E_{alive\ with\ control}} \quad (2)$$

  where $C_{treatment}$ is the average cost of smoking cessation treatment per patient, $C_{control}$ is the cost of control treatment. Costs of the intervention will be calculated based on unit costs of counselling meetings and nicotine replacement therapy. $E_{alive\ with\ treatment}$ and $E_{alive\ with\ control}$ are the percentage of patients who did not die during the 12 months of follow-up in the treatment group and in the control group respectively

For cost-effectiveness analyses, at first, hospital costs in both arms with and without costs of the tobacco treatment specialist (TTS) and nicotine replacement products will be compared per participant . Two simple linear regression model with the randomized treatment group as independent variable will be used to estimate the differences between treatment groups in terms of total hospital and treatment cost: one having the total hospital costs with addition of costs of the tobacco treatment specialist (TTS) and nicotine replacement products as dependent variable and another one having the total hospital costs without the costs of the tobacco treatment specialist (TTS) and nicotine replacement products as dependent variable. To evaluate the group difference in total hospital and treatment cost of the smoking cessation intervention versus control group the Wald test to check the significance of individual regression coefficients will be used. Normality will be checked using qq-plot and, if necessary, appropriate transformations of the dependent variables will be made.

At second, incremental cost per additional quit and incremental cost per additional life year saved of the preoperative institutional smoking cessation program compared to usual care will be calculated. Utilities will be calculated based on the Quality of life (SF-36 Index).

Analysis Sets to be used for: Full Analysis Set (FAS)

Cost-effectiveness analysis will be presented as follows:

Table 16 *Estimated means by treatment groups and estimated treatment effects along with their confidence intervals for total hospital cost, with and without costs of the tobacco treatment specialist (TTS) and nicotine replacement products, and the associated P-values from Wald Test*

Table 17 *Incremental cost-effectiveness ratio (ICER) for additional success in quitting smoking and for additional life year saved*

5.2.2 Subgroup Analysis

Collected variables

- Comprehensive Complication Index (Numeric) [Questionary 6]
- Cigarettes smoked per day in the past few days (Numeric Cigarettes/day) [Questionary 2]
- Patient date of birth (yyyy-mm-dd) [Questionary 1]
- Intermediate or high-risk procedure (Intermediate risk, high risk) [Questionary 1]
- Date of admission for surgery (yyyy-mm-dd) [Questionary 1]
- Date of the initial consultation (yyyy-mm-dd) [Questionary 4a]
- Date of counselling (yyyy-mm-dd) [Questionary 5]
- Last date of smoking cessation [Questionary 5]

Derived variables

- Whether the patient is $\leq$ 60 or >60 years old
- Time to surgery from first intervention/counselling = days between the date of the initial consultation and the date of admission for surgery
- Time to surgery from last quit smoking date = days between the last quit smoking date and the date of admission for surgery

To assess possible differences in CCI between the treatment groups taking into account the cigarettes smoked per day, after normality check using qq-plot, a multiple linear model will be used having the treatment group, the minimazation variables, cigarettes smoked per day and the interaction term between the treatment group and cigarettes smoked per day as independent variables and if necessary a transformation of the CCI will be applied. The same will be done using the time (days) to surgery from first intervention/counselling instead of cigarettes smoked per day as well as for the time (days) to surgery from the last quit smoking date. In addition, to evaluate differences in CCI in heavy smokers and light smokers, the choice of a cut-off like a mean split will be made if it is reasonable.
Ajustements of p-values for multiple testing will be made.

Analysis Sets to be used for: Full Analysis Set (FAS)

Subgroup analysis will be presented as follows:

Table 18 *The estimated effects of the treatment group, cigarettes smoked per day and the interaction term between the treatment group and cigarettes smoked per day will be presented along with their confidence intervals and P-values from Wald test to check the significance of individual regression coefficients.*

Table 19 *The estimated effects of the treatment group, days from first intervention/counselling to surgery, and the interaction term between the treatment group and days from first intervention/counselling to surgery will be presented along with their confidence intervals and P-values from Wald test to check the significance of individual regression coefficients.*

Table 20 *The estimated effects of the treatment group, days from the last quit smoking date to surgery, and the interaction term between the treatment group and days from the last quit smoking date to surgery will be presented along with their confidence intervals and P-values from Wald test to check the significance of individual regression coefficients.*

Figure 11 *A scatter plot for each level of the minimasation variables presenting the cigarettes smoked per day on the x-axis and the CCI on the y-axis by treatment group and showing the regression lines by treatment group.*

Figure 12 *A scatter plot for each level of the minimasation variables presenting the days from first intervention/counselling to surgeryy on the x-axis and the CCI on the y-axis by treatment group and showing the regression lines by treatment group.*

Figure 13 *A scatter plot for each level of the minimasation variables presenting the days from the last quit smoking date to surgery on the x-axis and the CCI on the y-axis by treatment group and showing the regression lines by treatment group.*

5.3 Missing data

To account for missing data after surgery took place multiple imputation will be used for the primary analysis.

5.4 Additional analyses

Collected variables

- Result of NicAlert Saliva test (negativ, 10-30 ng/ml, 30-100 ng/ml, 100-200 ng/ml, 200-500 ng/ml, 500-2000 ng/ml, >2000 ng/ml) [Questionary 8]
- Comprehensive Complication Index (Numeric) [Questionary 6]
- Date of admission for surgery (yyyy-mm-dd) [Questionary 9]
- Date of discharge from hospital after surgery (yyyy-mm-dd) [Questionary 9]
- Hospital costs for planned surgery (Numeric - CHF) [Questionary 9]
- Type of intraoperative complication (Code type of complication) [Questionary 6]
- Type of first post-operative complication (Code type of complication) [Questionary 6]
- Type of second post-operative complication (Code type of complication) [Questionary 6]
- Type of third post-operative complication (Code type of complication) [Questionary 6]
- Type of fourth post-operative complication (Code type of complication) [Questionary 6]
- Type of fifth post-operative complication (Code type of complication) [Questionary 6]

- Type of sixth post-operative complication (Code type of complication) [Questionary 6]
- Type of seventh post-operative complication (Code type of complication) [Questionary 6]
- Type of eighth post-operative complication (Code type of complication) [Questionary 6]
- Type of ninth post-operative complication (Code type of complication) [Questionary 6]
- Type of tenth post-operative complication (Code type of complication) [Questionary 6]
- Type of first complication treated in other institution (Code type of complication) [Questionary 6]
- Type of second complication treated in other institution (Code type of complication) [Questionary 6]
- Intraoperative complications (Yes/No) [Questionary 6]
- Severity grade of intraoperative complication (CDC Grade I, CDC Grade II, CDC Grade IIIa, CDC Grade IIIb, CDC Grade IVa, CDC Grade IVb, CDC Grade V) [Questionary 6]
- Clavien-Dindo classification of first post-operative complication (CDC Grade I, CDC Grade II, CDC Grade IIIa, CDC Grade IIIb, CDC Grade IVa, CDC Grade IVb, CDC Grade V) [Questionary 6]
- Clavien-Dindo classification of second post-operative complication (CDC Grade I, CDC Grade II, CDC Grade IIIa, CDC Grade IIIb, CDC Grade IVa, CDC Grade IVb, CDC Grade V) [Questionary 6]
- Clavien-Dindo classification of third post-operative complication (CDC Grade I, CDC Grade II, CDC Grade IIIa, CDC Grade IIIb, CDC Grade IVa, CDC Grade IVb, CDC Grade V) [Questionary 6]
- Clavien-Dindo classification of fourth post-operative complication (CDC Grade I, CDC Grade II, CDC Grade IIIa, CDC Grade IIIb, CDC Grade IVa, CDC Grade IVb, CDC Grade V) [Questionary 6]
- Clavien-Dindo classification of fifth post-operative complication (CDC Grade I, CDC Grade II, CDC Grade IIIa, CDC Grade IIIb, CDC Grade IVa, CDC Grade IVb, CDC Grade V) [Questionary 6]
- Clavien-Dindo classification of sixth post-operative complication (CDC Grade I, CDC Grade II, CDC Grade IIIa, CDC Grade IIIb, CDC Grade IVa, CDC Grade IVb, CDC Grade V) [Questionary 6]
- Clavien-Dindo classification of seventh post-operative complication (CDC Grade I, CDC Grade II, CDC Grade IIIa, CDC Grade IIIb, CDC Grade IVa, CDC Grade IVb, CDC Grade V) [Questionary 6]
- Clavien-Dindo classification of eighth post-operative complication (CDC Grade I, CDC Grade II, CDC Grade IIIa, CDC Grade IIIb, CDC Grade IVa, CDC Grade IVb, CDC Grade V) [Questionary 6]

- Clavien-Dindo classification of ninth post-operative complication (CDC Grade I, CDC Grade II, CDC Grade IIIa, CDC Grade IIIb, CDC Grade IVa, CDC Grade IVb, CDC Grade V) [Questionary 6]
- Clavien-Dindo classification of tenth post-operative complication (CDC Grade I, CDC Grade II, CDC Grade IIIa, CDC Grade IIIb, CDC Grade IVa, CDC Grade IVb, CDC Grade V) [Questionary 6]
- Clavien-Dindo classification of first complication treated in other institution (CDC Grade I, CDC Grade II, CDC Grade IIIa, CDC Grade IIIb, CDC Grade IVa, CDC Grade IVb, CDC Grade V) [Questionary 6]
- Clavien-Dindo classification of second complication treated in other institution (CDC Grade I, CDC Grade II, CDC Grade IIIa, CDC Grade IIIb, CDC Grade IVa, CDC Grade IVb, CDC Grade V) [Questionary 6]

Derived variables

- Length of hospital stay = days between the date of admission for surgery and the date of discharge from hospital after surgery, calculated for patients who are still alive
- Highest CDC = a variable showing for each patient the highest Grade of Clavien-Dindo complication observed (number, 1=CDC Grade I, 2=CDC Grade II, 3=CDC Grade IIIa, 4=CDC Grade IIIb, 5=CDC Grade IVa, 6=CDC Grade IVb, 7=CDC Grade V)
- Whether a patient was successfully discharged from hospital (no deaths in hospital) (Yes/No)

Differences in the result of NicAlert Saliva test between treatment group will be evaluated by applying Chi-square test in order to verify the hypothesis of no difference between treatment groups. In order to evaluate a possible linear correlation between the Comprehensive Complication Index and the length of hospital stay, the Pearson correlation index and the P-value from the Pearson correlation test will be presented after checking for normality using Shapiro-Wilk test and the qq-plot. If a violation to normality is present, a non-parametric correlation such as Spearman and Kendall rank-based correlation indexes and tests will be used. To investigate a possible non linear correlation, a Scatter plot and the nonlinear correlation estimate and adjusted p-value using adaptive spatial sampling with the "nlcor" function in R will be shown. The same will be done to evaluate a possible correlation between the Comprehensive Complication Index and the hospital cost, a possible correlation between highest CDC and the length of hospital stay and a possible correlation between highest CDC and the hospital cost.
For each Grade of Clavien-Dindo complication, the number of patients who had at least once specific Grade of Clavien-Dindo complication will be considered as the number of events of interest and used to calculate the absolute risk of having the specific Grade of Clavien-Dindo complication. The Absolute risk reduction (ARR) and, if possible, the Relative Risk (RR) between the treatment groups will be presented along their confidence intervals. The confidence intervals of the relative risk will be calculated using the normal approximation method. The same will be done for each type of complication that has occurred at least once.

Analysis Sets to be used for:
Table 21, 24, 25: Full Analysis Set (FAS)

Table 22, 23, 26, 27 and Figure 14, 15, 16, 17: Patients in the Full Analysis Set successfully discharged from the hospital (no death in the hospital).

Additional analyses results will be presented as follows:

Table 21 *Frequency of the results of NicAlert Saliva test between treatment groups and P-value from Chi-square test will be presented.*

Table 22 *Pearson correlation index or Spearman and Kendall rank-based correlation indexes between the Comprehensive Complication Index and the length of hospital stay along with the associated P-values from the most appropriate statistical test will be presented.*

Table 23 *Pearson correlation index or Spearman and Kendall rank-based correlation indexes between the Comprehensive Complication Index and the hospital cost along with the associated P-values from the most appropriate statistical test will be presented.*

Table 24 *A Forest plot showing for each Grades of Clavien-Dindo complication the Absolute risk reduction (ARR) or, if possible, the relative risk between the treatment groups along with confidence intervals.*

Table 25 *A Forest plot showing for each type of complication the Absolute risk reduction (ARR) or, if possible, the relative risk between the treatment groups along with confidence intervals.*

Table 26 *Pearson correlation index or Spearman and Kendall rank-based correlation indexes between the highest CDC and the length of hospital stay along with the associated P-values from the most appropriate statistical test will be presented.*

Table 27 *Pearson correlation index or Spearman and Kendall rank-based correlation indexes between the highest CDC and the hospital cost along with the associated P-values from the most appropriate statistical test will be presented.*

Figure 14 *A scatter plot presenting the length of hospital stay on the x-axis and the Comprehensive Complication Index on the y-axis along with the nonlinear correlation estimate and adjusted p-value.*

Figure 15 *A scatter plot presenting the hospital cost on the x-axis and the Comprehensive Complication Index on the y-axis along with the nonlinear correlation estimate and adjusted p-value.*

Figure 16 *A scatter plot presenting the length of hospital stay on the x-axis and the highest CDC on the y-axis along with the nonlinear correlation estimate and adjusted p-value.*

Figure 17 *A scatter plot presenting the hospital cost on the x-axis and the highest CDC on the y-axis along with the nonlinear correlation estimate and adjusted p-value.*

5.5 Harms

Adverse events will be reported annually in the periodic safety reporting (ASR) including name and date of complications, as well as severity (Clavien-Dindo classification). The primary outcome and events on which the sample size is based represents postoperative complications which includes SAEs. All SAEs within 90 days after surgery will be documented and analysed in this trial. As the causal relationship between the events and the intervention can be ruled out, any event assessment

regarding causality will not be performed.

Derived variables
Adverse Events (AEs): An adverse event is any untoward medical occurrence in a patient or a clinical investigation subject which does not necessarily have a causal relationship with the trial procedure. An AE can therefore be any unfavourable or unintended finding, symptom, or disease temporally associated with a trial procedure, whether or not related to it.

Serious adverse event (SAEs): A Serious Adverse Event (SAE) (ClinO, Art. 63) is any untoward medical occurrence that

- Results in death or is life-threatening
- Requires in-patient hospitalisation or prolongation of existing hospitalisation
- Results in persistent or significant disability or incapacity or
- Causes a congenital anomaly or birth defect

Analysis Sets to be used: Safety Analysis Set

Adverse events will be summarized by treatment group as follows:

Table 28 *Number of patients with at least one AE, number of patients with at least one serious AE, Number of AEs, Number of serious AEs, Number of deaths will be presented.*

Table 29 *Incidence of AEs by seriousness. A default frequency table will be presented.*

Table 30 *Incidence of AEs by severity. A default frequency table will be presented.*

In addition, AE data will be listed as follows:

Listing 1 *All AEs by-patient and within-patient by date of complications and severity.*

Listing 2 *Lists all patients with at least one serious adverse event.*

5.6 Statistical software and programming

The analysis will be carried out using R in combination with dynamic reporting.

6 Additional remark

This Statistical Analysis Plan has been written according to the guidelines for the content of Statistical Analysis Plans in Clinical Trials [8, 9].

7 References

[1] FSO SFSO. Swiss Health Survey 2017. 2017
[2] Lee SM, Landry J, Jones PM, Buhrmann O, Morley-Forster P. The Effectiveness of a Perioperative Smoking Cessation Program: A Randomized Clinical Trial. Anesth Analg. 2013;117:605-13.
[3] Møller AM, Villebro N, Pedersen T, Tønnesen H. Effect of preoperative smoking intervention on postoperative complications: a randomised clinical trial. Lancet. 2002;359:114-7.
[4] Lindström D, Sadr Azodi O, Wladis A, Tonnesen H, Linder S, Nasell H, et al. Effects of a perioperative smoking cessation intervention on postoperative complications: a randomized trial. Ann Surg. 2008;248:739-45.
[5] Thomsen T, Villebro N, Moller AM. Interventions for preoperative smoking cessation. Cochrane Database Syst Rev. 2014
[6] Slankamenac K, Graf R, Barkun J, Puhan MA, Clavien PA. The comprehensive complication index: a novel continuous scale to measure surgical morbidity. Ann Surg. 2013;258:1-7.
[7] Copeland GP, Jones D, Walters M. POSSUM: A scoring system for surgical audit. Br J Surg. 2005;78:355-60.
[8] https://jamanetwork.com/journals/jama/fullarticle/2666509
[9] https://www.equator-network.org/reporting-guidelines/guidelines-for-the-content-of-statistical-analysis-plans-in-clinical-trials/

This document was generated on Aprile 15, 2022 at 14:28.
